# The Presumed Influence of COVID-19 Misinformation on Social Media: Survey Research from Two Countries in the Global Health Crisis

**DOI:** 10.3390/ijerph18115505

**Published:** 2021-05-21

**Authors:** Yunjuan Luo, Yang Cheng

**Affiliations:** 1 Department of Online Communication, School of Journalism and Communication, South China University of Technology, Guangzhou 510006, China; yunjuan.luo@yahoo.com; 2 Department of Communication, North Carolina State University, Raleigh, NC 27695, USA

**Keywords:** COVID-19, health misinformation, social media, influence of presumed influence (IPI), China, USA

## Abstract

While the coronavirus 2019 (COVID-19) pandemic is spreading all over the world, misinformation, without prudent journalistic judgments of media content online, has begun circulating rapidly and influencing public opinion on social media. This quantitative study intends to advance the previous misinformation research by proposing and examining a theoretical model following an “influence of presumed influence” perspective. Two survey studies were conducted on participants located in the United States (N = 1793) and China (N = 504), respectively, to test the applicability of the influence of presumed influence theory. Results indicated that anger and anxiety significantly predicted perceived influence of misinformation on others; presumed influence on others positively affected public support in corrective and restrictive actions in both U.S. and China. Further, anger toward misinformation led to public willingness to self-correct in the U.S. and China. In contrast, anxiety only took effects in facilitating public support for restrictive actions in the U.S. This study conducted survey research in China and the U.S. to expand the influence of presumed influence (IPI) hypothesis to digital misinformation in both Western and non-Western contexts. This research provides implications for social media companies and policy makers to combat misinformation online.

## 1. Introduction

The coronavirus 2019 (COVID-19) is a disease outbreak caused by severe acute respiratory syndrome coronavirus 2 (SARS-CoV-2) [1]. Originally discovered in Wuhan, mainland China, in December 2019, this infectious illness has been evolving in more than 150 countries within just a few months, resulting in a global health crisis. Up until 11 April 2021, over 30,897,028 coronavirus cases were reported to the Centers for Disease Control and Prevention (CDC) and nearly 558,028 total deaths occurred in the United States, which has the most COVID-19 cases in the world [2]. More than 136 million cases have occurred globally, and over 2,945,771 lives have been lost worldwide [3]. When people face uncertainties due to this disease and continuously social distance, social media platforms provide a critical role in fulfilling diversified gratifications, such as information needs, social needs to connect with friends and families, and fun or enjoyment obtained from entertainment [4,5]. However, without prudent journalistic judgments of media content online, misinformation, referring to false and misleading information, began circulating rapidly on social media [6,7]. For instance, misinformation has made many people believe that using salt water or drinking bleach could kill the virus during the pandemic [8]. The general public, especially those who cannot easily identify misinformation and differentiate the misleading messages from health facts [9], might conduct misinformed behaviors, such as panic purchasing actions and mass meetings without proper protection. An updated search from O’Rear and Radvansky [10] also indicated that misinformation, compared to real news, could easily trigger negative emotions, such as anxiety and anger, and continued to influence public opinions even if corrective information had been released publicly.

As the world faced the threat of misinformation, the public from both Western and Eastern countries demonstrated different reactions during this pandemic. Countries in a Western context, such as the U.S., have the most COVID-19 cases, and people were still debating about controversial issues, such as wearing masks and reopening countries. Misinformation was circulating rapidly within a low-trust media environment, and many protestors still defied social distancing guidelines or regulations from the government. In contrast, China, in a non-Western context, first discovered the coronavirus and recognized the severity of the outbreak in January of 2020. After a lockdown of Wuhan and the governmental enforcement to counter misinformation on social media, the public generally stayed calm and accepted wearing a mask, staying at home, and reporting their temperatures via social media apps. Currently, the virus is being well-controlled in China. Situated within both Western and non-Western contexts, it is interesting to examine the U.S. and China in relation to the behavioral outcomes of interest (i.e., corrective and restrictive actions), given the differences between China and U.S. in the governments’ control over media and policies related to COVID-19. Public emotions and the presumed impact of COVID-19 misinformation from both countries, as the world’s two largest economies also deserve future exploration.

**Figure 1:**
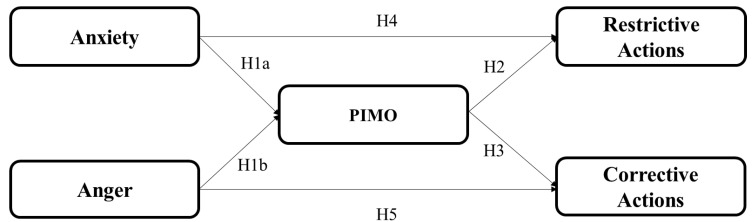
The Conceptual Framework. Note: PIMO = Presumed influence of misinformation on others.

This study, therefore, intends to advance the previous misinformation research by proposing and examining a theoretical model (as shown in Figure 1) following an “influence of presumed influence” perspective. This research is imperative for practitioners, who intend to combat misinformation and improve the efficiency of information management on social media. Deploying survey research in the U.S. (N = 1793) and China (N = 504), we focus on examining the relationships between anxiety and anger as negative emotions, the presumed influence of misinformation on social media, and behavioral intentions such as support for restrictive and corrective actions during a global health crisis. Objectives of this study include below dimensions. First, we intend to enrich the influence of presumed influence (IPI) theory in the area of digital misinformation. In this study, researchers examined the theoretical application of IPI in misinformation on a global health crisis and drew inferences from both Western and non-Western contexts. Second, this study extends previous literature about the emotional impact on public behavioral intentions [11,12] by exploring the associations between two distinct negative emotions (i.e., anxiety and anger) and their respective impact on public support for restrictive and corrective actions.

## 2. Literature Review

### 2.1. The Influence of Presumed Influence

According to Davison [13], people tend to overestimate the effects of media, and they believe that others are more influenced by media messages than themselves. Davison [13] also found that such other-self perceptual gaps would lead to behavioral changes such as the public disregard of health messages or individuals’ engagement in political activities.

Following this approach, numerous scholars (e.g., [14,15,16]) conducted research to examine and explain this interesting phenomenon in the past decades. Such scholars include Gunther and Storey [17], who presented their influence of presumed influence model as a comprehensive and general theory of media effects [17]. Gunther and Storey [17] asserted that the presumed media influence does not rely on self-other perceptual difference; instead, people infer their public opinions based on their perceptions of media messages and the presumed media influence on others. After, they would “react to that perception of influence” [17] (p. 201). Consequently, the IPI model assumes an indirect effect of mass media: people’s perceived influence of a message on others affects attitudes and behavioral outcomes of individuals. For example, Hong and Kim [18] conducted research among college students and explored how the presumed media impact on others would influence their health prevention behaviors, such as safe sex and skin cancer prevention. Their results supported the indirect effect of mass media, confirming that the presumed media influence played an important role in communicating health information. Hoffner and Cohen [19] launched an online survey to examine 198 adults’ perceived influence of news reports on the Virginia Tech shootings. Their findings supported the positive association between the perceived influence of the TV series Monk on others and the willingness to disclose mental health treatment, among the group of participants without prior experience with mental illness. Shin and Huh [20] found parents believed that others (i.e., children) were vulnerable to video games. Thus, parents projected that such media platforms are powerful, and they must prevent their children from playing games. Previous literature also examined the IPI model and supported its propositions about diversified media content, including election news [21], female’s use of magazines [22], corporate recall misinformation [23], pro-smoking messages [24], and blog messages about contaminations in fish [11], etc.

### 2.2. Presumed Impact of Misinformation

Within the limited discussion on the persistent negative influence of misinformation, scholars (e.g., [7,23,25,26,27]) in the field of health and digital communication began to investigate individuals’ perceived impact of misleading information online. Liu and Huang [26], for instance, published their latest article about the third-person effects of fake news about COVID-19. Their data collected from China indicated that the perceptual gap existed when participants tended to believe that the influence of fake news would exert a higher impact on others than on themselves [26]. However, this study was only located in China (i.e., a non-Western context), and it did not consider the presumed influence of misinformation on others (PIMO) as the research focus.

Additionally, the antecedents and outcomes of PIMO remained unexplored as well. In Cheng and Chen’s [23] research, their results explained why individuals believed that negative news would project the influence of a powerful media on others, and what kind of behavioral intentions might occur due to presumed influence on others (cf. [17]). However, their study only emphasized corporate misinformation within a business context, and misinformation as a probable threat to global public health was ignored. More importantly, as previous literature indicated, emotions presented their unique contributions to the publics’ attitudes and behaviors during H1N1 pandemic flu (e.g., [12]), election events (e.g., [28,29]), the Fukushima nuclear accident [11], and Y2K technological crisis [30]. However, how different types of negative emotions, such as anxiety and anger, influence the PIMO and further affect behavioral outcomes during the COVID-19 pandemic deserves further investigations.

### 2.3. Negative Emotions: Anxiety and Anger

Emotions are generally regarded as “internal, mental states representing evaluative, valenced reactions to events, agents, or objects that vary in intensity” [31] (p. 163). When people encounter a stimulus, they may develop different varieties of emotions, such as anxiety, anger, sadness, pride, or gladness, etc. In the context of the COVID-19 crisis, exposure to misleading news information of the disease could easily provoke negative affective responses such as anxiety due to uncertainties and anticipated danger or anger toward parties who should be responsible for harm [26].

Each of the above-mentioned negative emotions (i.e., anxiety or anger) has its own predecessors [32]. Anxiety arises when people feel they have little control over an external threat under conditions of ambiguity [33,34]. People experience anxiety due to “relationship, lack of confidence, aimless future, work incompetence, financial concerns, and socio-political concerns” [33] (p. 103). In contrast, anger is evoked when harm or loss is “blamed on another person” [35] [p. 218) and intensified when people feel misled and unjustly hurt by others [34,36]. In this study, we focused on the above-mentioned two typical negative emotions (i.e., anxiety and anger) that participants might develop toward misinformation within the COVID-19 crisis.

According to Schwarz [37], individuals might experience affective heuristic, such as negative or positive moods, first, and then conduct cognitive judgments, such as satisfaction, with products or goal incongruence with media messages. Scholars such as Lee, Scheufele, and Lewenstein [38] and Izard [39] also supported the relationships between affective responses and cognitive evaluations. Regarding the impact of negative emotions aroused by media information and the presumed influence on others, the current literature is limited. For instance, Wei, Lo, and Golan [40] studied the presumed influence of media coverage of China in the U.S. and found that negative emotions, such as anger, could positively influence the presumed media influence on others. Meanwhile, the past research on misinformation (e.g., [15,26]) does not provide thorough evidence on the role of negative emotions in the IPI model, especially under the circumstance of a global pandemic within both Western and non-Western countries.

To enrich previous discussions, this study hypothesizes that negative emotions such as anxiety or anger toward misinformation will motivate people to evaluate and judge the negative outcomes that such media content might bring for others within the same pandemic in society. The higher level of negative emotions, such as anxiety and anger, individuals might possess, the more likely they would consider the social undesirability of such misinformation toward others during this COVID-19 crisis. Consequently, we propose H1a and H1b as follows.

**Hypothesis** **1** **(H1).** *Anxiety (H1a) and anger (H1b) toward COVID-19 misinformation will be positively related to the presumed media influence on others.*

### 2.4. Restrictive Actions

Previous IPI research has long been devoted to exploring behavioral intentions that may arise out of the presumed media influence on others. Most studies have largely focused on two major behavioral consequences: restrictive actions, such as support for governmental regulation or censorship [23] and withdrawal from public discussions [41]; Corrective actions such as the participation in political discussions [42] or conduction of expressive behavior, such as political talks [43].

Restrictive actions have been widely examined as one type of behavioral intentions of presumed media influence [44]. Previous theory suggests that people’s overestimation of perceived harmful influence of media messages on others leads them to react in favor of restrictive regulations from the government in hopes of mitigating the predicted unfavorable effects on others [13,45]. A large body of studies has empirically tested the relationship between support for restrictive actions and perceived influence of socially undesirable media content, such as pornography [46], television violence [47], and negative political advertising [48]. In a recent study on violent video games, Liu, Lo, and Wei [49] found that people’s presumed influence of such games on other players was a significant predictor of their support for restrictive actions to prevent the potential harms of violent video games. Using a meta-regression approach, Chung and Moon [14] further supported that presumed influence on others was a stronger influencer of censorship attitudes than self-other perceptual differences.

Building on previous studies on the relationship between the presumed influence of others and restrictive actions, we proposed H2.

**Hypothesis** **2** **(H2).** *Presumed influence of misinformation on others would influence people’s willingness to support restrictive actions.*

### 2.5. Corrective Actions

Although research on the outcomes of perceived media influence largely focused on restrictive actions [15], the public support for corrective actions, as another key consequence of presumed influence, have also been extensively examined in previous studies [40,44,50]. Lim and Golan [14] examined the perceived influence of political parody videos on social media platforms and found a significant relationship between perceived influence on others and individuals’ willingness to engage in social media activism as a form of corrective action. Barnidge and Rojas [44] tested the corrective action hypotheses and found that the presumed influence of biased media motivated people to participate in political talk more frequently in an attempt to correct those perceived biases. In a corporate business context, Cheng and Chen [23] also found that the presumed influence of fake news on others was positively related to support for corporate corrective actions. Based on results from past literature, we thus proposed H3 below:

**Hypothesis** **3** **(H3).** *Presumed influence of misinformation on others would affect people’s willingness to support corrective actions.*

### 2.6. Anxiety, Anger, and Behavioral Intentions

According to Mehrabian–Russell [51], a stimulus in a given situation can trigger a certain state of emotion, which subsequently may motivate individuals to take adaptive behaviors. Previous literature has provided evidence that message-induced negative emotions positively affected the public support for restrictive actions. For example, anxiety commonly produces an over-evaluation of threats and leads to avoidance or escape [34,52]. An empirical study from Kim (2015) on news coverage of political voting results indicated that those who were anxious would prefer to support the government restrictions on voting information in order to avoid the occurrence of negative outcomes. Valentino et al. [52] also found that anxiety could lead to less powerful and costly political participation activities. Given the fact that anxiety is commonly linked to escaping or restrictive behavioral intentions during the COVID-19 crisis [26], we proposed H4 as follows.

**Hypothesis** **4** **(H4).** *Anxiety toward COVID-19 misinformation will positively predict the public support for restrictive actions.*

Previous literature also provided evidence that message-induced negative emotions positively influenced the support for corrective actions. For example, Wei et al. [42] suggested that negative affections elicited by U.S. news about China led to the public support for the Chinese government’s campaign in an effort to correct unflattering news coverage of China. Jin, Fraustino, and Liu [53] supported that the publics’ negative emotions would trigger their ongoing coping behaviors in crisis situations.

Furthermore, according to Huddy et al. [34], all negative emotions should be associated with the avoidance of danger or harm, but the model does not account for the distinction among different types of negative emotions. In fact, anger follows a behavioral approach that leads to the diminishment of perceived risks and problem-focused coping behavior [34,52], which is distinguishable with the avoiding or restriction actions that anxiety might elicit through media messages. For instance, the study by Valentino et al. [52] demonstrated that participants who experienced anger showed a greater intention to engage in political actions than those who contained anxiety. In a study of environmental issues, Nerb and Spada [54] also suggested that anger evoked by cognitive evaluation of environmental risks was positively linked to the corrective action of boycotting. In a crisis context, Gault and Sabini [55] indicated that anger motivated people’s supportive actions for punishing the responsible organizations. In the political context, Kim’s study [28] presented that anger was a positive predictor of voters’ political participation intention to counter the perceived bias in reported election poll results against their preferred candidate. Thus, during the COVID-19 crisis, we hypothesize that those who experience anger toward misinformation about the disease would be more likely to take corrective actions.

**Hypothesis** **5** **(H5).** *Anger toward COVID-19 misinformation will be positively associated with corrective actions.*

To examine the above-mentioned five sets of hypotheses proposed in the conceptual model (as shown in Figure 1), we collected data sets in both the United States and China. Below we present methods and findings of these two studies, respectively.

## 3. Study I: The United States

### 3.1. Method

We designed the survey study on Qualtrics in February. Upon the approval from the Institutional Review Board from a public university located in the southwest of the U.S., we collected data from 3 April 2020 to 10 April 2020. Panel responses were purchased from Lucid, an online sampling company located in the United States. This online panel has been frequently adopted by past researchers [56] and contains several procedures, such as blocking bots, screening participants via attention checkers and open-ended questions, and setting up quality programs and fraud prevention services to ensure high-quality research data [57]. Invitations were sent to nearly 2000 participants based on percentages of age, gender, education, ethnicity, and income in the national population. A total of 1793 (To ensure a representative population, we calculated the sample size based on the predicted population (331,002,651 in the U.S. in 2020) [58]. Assuming a 95% confidence level and 5% precision (margin of error), the minimum sample size needed was 272 adults from the U.S.) respondents successfully completed the questionnaire, and each of them was shown the definition and examples of misinformation at the beginning of the study.

**Participants.** Of the sample from the U.S., the gender ratio is 43% male versus 57% female. The average age was 48 years, with 30.1% falling between the range from 18 to 34, 27.6% between 35 and 54, 36.5% between 55 and 74, and 5.8% were above 75. Among the 1793 respondents, 77.2% identified themselves as Caucasian/White, 9% reported themselves as Black or African American, 6.2% were Latino/Hispanic, 4.8% were Asian American/Pacific Islander, 0.9% were American/American Indian, and 1.9% were others. Regarding education level, 40.5% had received their bachelor’s degree or associate degree, 21.8% had received some college or no degree, 18.6% had their high school diploma or general educational development, 17% had a master or doctoral degree, and 2.1% had less than high school diploma. In terms of annual income per household, 42.5% reported that they had an annual household income of $40,000 (USD) or under; 17.3% more than $100,000; 17.2% between $40,001 and $60,000, 14.3% between $60,001 and $80,000, and 8.7% between $80,001 and $100,000.

**Measures.** To examine anxiety and anger, as two distinguished negative emotions, this study reviewed previous scales [28,59] and asked participants to answer three basic questions for their emotion of anxiety, such as “When you encounter misinformation about COVID-19, to what extent do you feel: anxious/worried/nervous?” (α = 0.95) and three items for the emotion of anger, such as “When you encounter misinformation about COVID-19, to what extent do you feel: angry/outraged/annoyed?” (α = 0.89). A five-point Likert scale was applied for both questions, which ranged from “not at all” = “1” to “a great deal” = “5”.

*Presumed influence of misinformation on others (PIMO)*. To measure the presumed media influence on others, we applied five items (α = 0.86) from previous scales [15,23]. The sample questions included, “I believe other people are very concerned about the spread of COVID-19 misinformation on social media”, “I believe that misinformation misleads others’ preventive actions against COVID-19”, and “I believe other people are very concerned about the authenticity of COVID-19 news that they receive on social media.” All items used a five-point Likert-type scale, ranging from “strongly disagree” = “1” to “strongly agree” = “5”.

*Corrective actions.* Based on Lim’s scale [50], we applied three items following a five-point Likert scale to measure the public support for self-corrective activities (α = 0.79). Questions such as “I would check the authenticity of the misinformation message before I forward it”, “When I detect misinformation on social media, I would report it to the platform”, and “When I detect misinformation on social media, I would place a complaint against its author” were asked.

*Restrictive actions.* To measure the public support for restrictive actions (α = 0.87), we consulted Cheng and Luo’s study [15] and adopted four questions for examination. A sample item was “I would support that misinformation should be blocked/censored by social media platforms”. Ranging from “strongly disagree” = “1” to “strongly agree” = “5”, a five-point scale was applied.

### 3.2. Results

**Descriptive data.** In this study, we first presented descriptive data of each variable and adopted “low (1.00–1.99)”, “moderately low (2.00–2.99)”, “neutral (3)”, “moderately high (3.01–3.99)”, and “high (4.00–5.00)” as ranges of values. As shown in the Appendix A, data showed participants in the U.S. has a moderately low level of anxiety (M = 2.85, *SD* = 1.41) toward misinformation. In contrast, respondents contained a moderately high level of anger (M = 3.64, *SD* = 1.27) toward misinformation, perceived influence of misinformation on others (M = 3.82, *SD* = 0.85), and support for corrective actions (M = 3.69, *SD* = 1.19). Meanwhile, the public support for restrictive actions reached a high level (M = 4.09, *SD* = 0.98).

**Hypotheses testing.** In predicting the presumed influence of misinformation and public support for restrictive and corrective actions, we conducted data analyses on SPSS 20.0 (SPSS Inc., Chicago, IL., USA). We measured and controlled demographic variables, such as gender, age, education, household income, and exposure to misinformation (i.e., we asked participants how often they have encountered misinformation on social media platforms, such as Twitter, Facebook, or WeChat, etc.) in data analysis, as previous literature demonstrated these variables might be associated with the presumed media influence on others and public behavioral intentions toward negative messages (e.g., [26,42]).

To test H1, regarding the relationships between negative emotions, such as anxiety, anger, and the PIMO, we conducted the hierarchical regression analysis. Three blocks were entered into SPSS 20, starting from demographic variables in block 1, then media exposure in block 2, and negative emotions in block 3. Data show that in the U.S., both anxiety (*β* = 0.17, *p* < 0.001) and anger (*β* = 0.14, *p* < 0.001) were positively related to the PIMO, supporting both H1a and H1b. This demonstrates that the more likely individuals possess anger or anxiety toward misinformation about COVID-19, the higher level of perceived negative impact of misinformation they will perceive towards other people in society during this pandemic. Table 1 also shows that in the U.S., exposure of misinformation significantly predicts the PIMO.

In predicting the public support for restrictive or corrective actions from H2 to H5, this study again applied the hierarchical regression analyses. Four blocks were entered into the analysis step by step, starting from demographic variables, then media exposure in block 2, negative emotions in block 3, and presumed influence in the last block. Table 2 shows that in the United States, presumed influence of misinformation on others could significantly predict both restrictive (*β* = 0.37, *p* < 0.001) and corrective actions (*β* = 0.33, *p* < 0.001). This means that the influence of the presumed influence of misinformation could enhance the public support for governmental restrictions of misinformation on social media, and meanwhile facilitate the public support for self-corrective actions to recognize or report misinformation. Consequently, both H2 and H3 were supported.

Data also indicated that negative emotions were the most important block, explaining the largest percentage of variance in the regression analysis. Anxiety was positively associated with restrictive actions (*β* = 0.06, *p* < 0.01), supporting H4; Anger exerted a direct and positive impact on corrective actions (*β* = 0.18, *p* < 0.001), supporting H5.

Demographically, it is interesting to notice that age may work as a positive predictor of the public support for restrictive actions (*β* = 0.10, *p* < 0.001); while generating a negative impact on corrective actions (*β* = −0.11, *p* < 0.001). Thus, young adults may tend to be against governmental control or censorship of misinformation on social media and they prefer to conduct self-corrective activities. In contrast, the older generation in the U.S. may prefer the central control of online information from governments.

**Indirect effects.** As previous literature [28,29] indicated, negative emotions such as anxiety and anger might indirectly affect the behavioral intentions as well via the presumed media influence. We thus performed mediation tests with a bias-corrected bootstrapping procedure (*N* = 5000 samples) in Amos 20 to test the indirect effects between these variables. Results indicated that indirect effects between anxiety and restrictive actions (*β* = 0.08, *p* < 0.001 (BC 95% CI: 0.06 to 0.10)); anger and corrective actions (*β* = 0.06, *p* < 0.001 (BC 95% CI: 0.04 to 0.09)) were significant. PIMO thus was a partial mediator for the relationships between anger, anxiety, and behavioral intentions.

## 4. Study II: China

### 4.1. Method

To investigate the above-mentioned hypotheses in China, we first translated all questions in Study I from English to Chinese, and back-translated those items to ensure the consistency of questions measured in the studies between the U.S. and China. At the beginning of the survey, all participants were presented with the definition and an example of misinformation during the COVID-19 pandemic. Following the background information, we asked people’s perceptions and behavioral intentions and provided attention-checking questions to ensure the quality of this online survey. Second, we conducted a pilot test among local residents and further checked the accuracy and applicability of this Chinese-version questionnaire with the local context. Third, to enroll participants in mainland China, we employed Sojump, one of the professional local survey companies [49] as the survey platform, which comprised 2 million Chinese panel members. A total of 504 (To ensure a representative population, we calculated the sample size based on the predicted population (1,439,323,776 in China in 2020) [60]. Assuming a 95% confidence level and 5% precision (margin of error), the minimum sample size needed was 281 adults from China.) respondents participated in and completed this study.

**Participants.** Among the 504 participants enrolled in China, 31.9% were male and 68.1% were female, with an average age of 33 years, with 57.3% falling between the range from 18 to 34, 40.9% between 35 and 54, and 1.8% between 55 and 70. Regarding education level, 62.9% had received their bachelor’s degrees, 11.1% completed some college or received their associate degree, 10.9% reported they had not received their high school diploma, 8.3% had their high school diploma or general educational development, and 6.7% had a master or doctoral degree. In terms of annual income per household, 51.4% reported that they had an annual household income of 12,000 yuan (CNY) ($1824) or under; 25.4% between 36,001 ($5455) and 96,000 yuan ($14,545), 12.3% above 96,000 yuan ($14,545), and 11% between 12,001 (1818) and 36,000 yuan ($5455).

**Measures.** All items applied a five-point Likert-type scale, which ranged from “strongly disagree” = “1” to “strongly agree” = “5”; “not at all” = “1” to “a great deal” = “5”. In Study II, we deployed the same measures of variables, such as anger, anxiety, presumed influence of misinformation on others, and restrictive and corrective actions. The Cronbach’s alpha coefficients all items reached a satisfactory level of reliability, ranging from 0.80 to 0.93.

### 4.2. Results

**Descriptive data.** Following study I, we continued using “low (1.00–1.99)”, “moderately low (2.00–2.99)”, “neutral (3)”, “moderately high (3.01–3.99)”, and “high (4.00–5.00)” as ranges of values to describe all variables. Data demonstrated that residents in mainland China have a moderately high level of anger (M = 3.41, *SD* = 0.90) and anxiety (M = 3.01, *SD* = 0.92) toward misinformation on the Chinese social media. Meanwhile, participants presumed a moderately high level of influence of misinformation on others (M = 3.68, *SD* = 0.64), and support for corrective actions (M = 3.66, *SD* = 0.73). In addition, respondents reported a high level of public support for restrictive actions toward misinformation (M = 4.16, *SD* = 0.81).

**Hypotheses testing.** To test the proposed hypotheses, we employed hierarchical regression analyses again based on data collected in mainland China. Demographic variables and media exposure served as control variables in early blocks entered into the SPSS 20 software.

Results from Table 1 depicted that in the Chinese society, both anxiety (*β* = 0.10, *p* < 0.05) and anger (*β* = 0.23, *p* < 0.001) were positively associated with the PIMO, supporting H1.

To examine the relationships between negative emotions, presumed influence, and the public support for restrictive or corrective actions in China, we again applied the hierarchical regression analyses. Data from Table 2 demonstrate that, in China, the PIMO could significantly predict both restrictive (*β* = 0.21, *p* < 0.001) and corrective actions (*β* = 0.20, *p* < 0.001), supporting both H2 and H3. Meanwhile, negative emotions serve as the most important block, explaining 8% and 11% variance in both regression analyses. Anxiety failed to predict the public support for restrictive actions (*β* = −0.05, *p* = 0.29), thus H4 was not supported based on the dataset collected in China. Anger contained a direct and positive influence on corrective actions (*β* = 0.28, *p* < 0.001), supporting H5.

Demographically, it is noted that age has a positive and direct influence on the public support for restrictive actions (*β* = 0.14, *p* < 0.05), which means that the younger the individuals are in mainland China, the more likely they won’t support the governmental restrictive actions toward social media.

**Indirect effects.** We again examined the indirect effects via conducting mediation tests with a bias-corrected bootstrapping procedure (*N* = 5000 samples) in Amos 20. Results indicated that the indirect effect between anger and corrective actions (*β* = 0.05, *p* < 0.001 (BC 95% CI: 0.02 to 0.10)) was significant. Anxiety could not significantly predict restrictive actions (*β* = 0.01, *p* = 0.13 (BC 95% CI: −0.01 to 0.05)). PIMO, therefore, was a full mediator for the association between anxiety and restrictive actions in China.

## 5. Discussion

Expanding the influence of presumed influence (IPI) theory on digital misinformation within a global health crisis, this study investigated the relationships between two types of negative emotions (i.e., anger and anxiety) toward misinformation on COVID-19, the PIMO, and public support for restrictive and corrective actions. Findings from both survey studies of 1793 respondents in the U.S. and 504 participants in mainland China suggest that, overall, individuals perceive others to be vulnerable to the impact of misinformation of COVID-19 on social media. Also, anger and anxiety significantly predicted perceived influence of misinformation on others; presumed influence on others positively affected public support in corrective and restrictive actions in both the U.S. and China. Furthermore, anger toward misinformation led to public willingness to self-correct in the U.S. and China. In contrast, anxiety only took effect in facilitating public support for restrictive actions in the U.S, rather than in China. Theoretical and practical implications of these results are discussed below.

First, this study contributes to the theoretical advancement of the influence of presumed influence (IPI) theory in a crisis context and expands this theory to digital misinformation in both Western and non-Western contexts. Previous IPI literature scarcely focuses on public health issues and is limited in one single study context [11,46]. When the misinformation of COVID-19 disease is intensifying on social media from all over the world [8], it is urgent to understand the public perception of misinformation proliferation toward this global pandemic and its behavioral outcomes in societies. By linking negative emotions, PIMO, and public support for restrictive and connective actions in one theoretical framework, we studied both antecedents and outcomes of perceived media influence on others in crisis situations. Results indicated that regardless of individuals’ cultural backgrounds, people perceived others as highly impacted by misinformation during this pandemic, which supported the IPI theory in both U.S. and China. Furthermore, in both countries, participants who contained a high level of negative emotions were more likely to perceive the PIMO, and such PIMO further facilitated the public support for both restrictive and corrective actions toward COVID-19 misinformation, supporting and enriching evidence from previous literature (e.g., [23]).

Second, results of this research extend previous discussions on the impact of negative emotions in the IPI literature. According to Lang [61] and Lang et al. [62], one’s emotional response is crucial in determining the amount of cognitive resources that can go into message elaboration. The functional theorists also suggested that emotions affect individuals’ cognitive and perceptual processes [39]. Negative emotions, such as fear and anxiety, can affect information processing, resulting in attitudinal changes [31]. For instance, scientific information framed with negative affections differed from non-affection messages in terms of their media impact on the public perception of climate change [63]. However, previous IPI literature on the relationship between negative emotions aroused by media messages and inferred effects of media on others generated limited results. Due to varying quality and representativeness of samples collected from different contexts, scholars such as Hoffner, Fujioka, Cohen, and Seate [64] generated inconsistent findings and did not support the association between negative emotions and the presumed influence of media coverage about mass shootings on others. This study thus filled the gap by showing that negative emotions elicited by misinformation on COVID-19 likely functioned as a cognition mechanism [40] and motivated people to evaluate deeper about the impact of such media messages on others’ health conditions in both U.S. and China.

Furthermore, we accounted for the distinction among different types of negative emotions [34] and their associations with restrictive and corrective actions, respectively. Findings of this study supported that the action tendencies of anxiety conform to this avoidance pattern in the U.S., as a Western context, but those of anger follow the behavioral approach in both U.S. and China. Interestingly, in the Chinese context, anxiety could not significantly predict the restrictive actions, and the PIMO fully mediated the relationship between anxiety and restrictive actions. A potential explanation of these results is, different from participants in a Western context, respondents in the non-Western Chinese society valued the collectivist culture and assumed “we” as a group, thus the high level of presumed influence of negative messages on others could take an important role in influencing the public support for restrictive actions [42]. It is also noted that in Chinese society, education as a demographic variable could significantly influence the public support for corrective actions. This could be explained that in mainland China, highly educated people might contain a high level of self-efficacy to identify misinformation [23], and then they were more likely to take actions to help stop COVID-19 misinformation.

Third, this research provides implications for social media companies and policymakers to combat misinformation online. Results, for instance, indicate the importance of the presumed influence of misinformation, which leads to both restrictive and corrective actions combating misinformation in both contexts. When false information regarding health issues was regarded as a probable threat to public health [6], this study suggested that practitioners could emphasize the negative impact of misinformation on others including friends and family members, and motivate individuals’ engagement on both restrictive and corrective actions. This research also demonstrated that different types of negative emotions, such as anger and anxiety, have distinct effects on behavioral intentions that are consistent with specific emotions. Consequently, when the public fuels negative emotions toward COVID-19 misinformation on social media, practitioners might notice that different emotional states can affect subsequent perceptions and behaviors [35,65]. For example, anxiety toward misinformation might lead to avoiding behavior, while anger could trigger the self-corrective behavior among individuals.

Regarding further implications for public health and pandemic management, this study suggested that policy makers could emphasize the presumed negative influence of COVID-19 misinformation on others and utilize it as an effective communication strategy to enhance the public support for restrictive and corrective actions toward such misinformation. As long as the actual public engagement on the COVID-19 facts could be increased, more and more individuals might conduct health prevention behavior such as mask-wearing and hand-washing. Consequently, the public disregard of pandemic policies could be prevented and the potentially harmful impact of misinformation on morbidity and mortality could be reduced as well.

## 6. Limitations

This research contains several limitations. First, we conducted cross-sectional survey research in both countries, which did not adopt a mixed-methods approach. Future studies might conduct both qualitative and quantitative studies following a parallel triangulation strategy to re-examine how anger and anxiety exert different impacts on the public support for restrictive or corrective actions toward misinformation during the COVID-19 pandemic. Second, this study only focused on two main types of negative emotions, which did not consider positive emotions such as pride and hope, future studies could compare negative and positive emotions and their influences on the perceived influence of misinformation on others. Finally, yet importantly, this article did not discuss minorities or low-income and -educated people, who might deserve future research attention.

## 7. Conclusions

This study conducted survey research in China and the U.S. to expand the IPI hypothesis to digital misinformation in both Western and non-Western contexts. Findings demonstrated the distinction among different types of negative emotions and their associations with restrictive and corrective actions, respectively. This research provides implications for social media companies and policymakers to combat misinformation online. Future experimental studies might be conducted to examine the impact of emotions and presumed media influence on the public support for restrictive or corrective actions toward COVID-19 misinformation.

## Figures and Tables

**Table 1:** Hierarchical Regression Analysis Predicting the Presumed Influence of Misinformation on Others (PIMO) in both U.S. and China.

Independent Variables	PIMO_U.S._		PIMO_China_	
	Beta	*p*-Value	Beta	*p*-Value
**Block 1: Demographics:**				
Gender	−0.04	0.12	0.02	0.72
Age	0.05 *	0.05	−0.03	0.66
Education	−0.04	0.15	−0.03	0.58
Income	−0.01	0.85	0.09	0.12
△R^2^	0.00		0.01	
**Block 2: Media Exposure:**				
Exposure of misinformation	0.16 ***	<0.001	0.03	0.46
△R^2^	0.05		0.00	
**Block 3: Negative Emotions:**				
Anxiety	0.17 ***	<0.001	0.10 *	0.04
Anger	0.14 ***	<0.001	0.23 ***	<0.001
△R^2^	0.07		0.08	
Total adjusted R^2^	0.12		0.08	

Notes: Beta weights and *p*-values are from the final regression equation with all blocks of variables in the model. Gender was recorded as follows: 1 = male, 2 = female. *** *p* < 0.001; * *p* < 0.05.

**Table 2:** Hierarchical Regression Analysis Predicting Support for Restrictive and Corrective Actions in both U.S. and China.

Independent Variables	Restrictive Actions_U.S._		Corrective Actions_U.S._		Restrictive Actions_China_		Corrective Actions_China_	
	Beta	*p*-Value	Beta	*p*-Value	Beta	*p*-Value	Beta	*p*-Value
**Block 1: Demographics:**								
Gender	0.02	0.48	−0.04	0.08	0.07	0.10	0.02	0.59
Age	0.10 ***	<0.001	−0.11 ***	<0.001	0.14 *	0.02	−0.00	0.98
Education	−0.02	0.44	0.02	0.45	0.03	0.53	0.11 *	0.02
Income	00.02	0.43	0.01	0.88	0.08	0.19	0.03	0.62
△R^2^	0.02		0.02		0.04		0.01	
**Block 2: Media Exposure:**								
Exposure of misinformation	−0.01	0.60	0.06 **	0.005	−0.07	00.11	−0.08	0.65
△R^2^	0.02		0.03		0.00		0.00	
**Block 3: Negative Emotions:**								
Anxiety	0.06 **	0.006	0.12 ***	<0.001	−0.05	0.29	0.01	0.83
Anger	0.25 ***	<0.001	0.18 ***	<0.001	0.24 ***	<0.001	0.28 ***	<0.001
△R^2^	0.13		0.11		0.08		0.11	
**Block 4: Presumed influence:**								
**Perceived influence on others**	0.37 ***	<0.001	0.33 ***	<0.001	0.21 ***	<0.001	0.20 ***	<0.001
△R^2^	0.12		0.10		0.04		0.04	
Total adjusted R^2^	0.28		0.26		0.16		0.15	

Notes: Beta weights and *p* values are from the final regression equation with all blocks of variables in the model. Gender was recorded as follows: 1 = male, 2 = female. *** *p* < 0.001; ** *p* < 0.01; * *p* < 0.05.

## Data Availability

The data are not publicly available due to privacy or ethical restrictions.

## Appendix A

**Table A1:** Survey Questions and Descriptive Data in Studies.

Variables	Measurement Item
**Presumed influence of misinformtion on others (PIMO)** (M_U.S._ = 3.82, *SD*_U.S._ = 0.85) (M_China_ = 3.68, *SD*_China_ = 0.64)	I believe other people are very concerned about the spread of COVID-19 misinformation on social media.
	I believe that misinformation misleads others’ preventive actions against COVID-19.
	I believe other people are very concerned about the authenticity of COVID-19 news that they receive on social media.
	I believe misinformation misleads other people’s understanding of COVID-19.
	I believe other people are very concerned about authenticity of COVID-19 news that they share/retweet on social media.
**Anxiety** (M_U.S._ = 2.85, *SD*_U.S._ = 1.41) (M_China_ = 3.01, *SD*_China_ = 0.92)	When you encounter misinformation about COVID-19, to what extent do you feel________?
	Nervous
	Worried
	Anxious
**Anger** (M_U.S._ = 3.64, *SD*_U.S._ = 1.27) (M_China_ = 3.41, *SD*_China_ = 0.90)	When you encounter misinformation about COVID-19, to what extent do you feel________?
	Angry
	Outraged
	Annoyed
**Corrective actions** (M_U.S._ = 3.69, *SD*_U.S._ = 1.19) (M_China_ = 3.66, *SD*_China_ = 0.73)	When I detect misinformation on social media, I would report it to the platform.
	When I detect misinformation on social media, I would place a complaint against its author.
	I would check the authenticity of the misinformation message before I forward it.
**Restrictive actions** (M_U.S._ = 4.09, *SD*_U.S._ = 0.98). (M_China_ = 4.16, *SD*_China_ = 0.81).	Accounts who post misinformation on social media should be removed.
	I would support legislation to prohibit the spread of misinformation on social media.
	I would support that misinformation should be blocked/censored by social media platforms.
	I would sign an online petition demanding the government to contain the spread of misinformation.
